# Cryptogenic Organizing Pneumonia: Evolution of Morphological Patterns Assessed by HRCT

**DOI:** 10.3390/diagnostics10050262

**Published:** 2020-04-29

**Authors:** Francesco Tiralongo, Monica Palermo, Giulio Distefano, Ada Vancheri, Gianluca Sambataro, Sebastiano Emanuele Torrisi, Federica Galioto, Agata Ferlito, Giulia Fazio, Pietro Valerio Foti, Letizia Antonella Mauro, Carlo Vancheri, Stefano Palmucci, Antonio Basile

**Affiliations:** 1 Radiology Unit 1, Department of Medical Surgical Sciences and Advanced Technologies “GF Ingrassia”—University Hospital “Policlinico-Vittorio Emanuele”, University of Catania, 95123 Catania, Italy; 2 Regional Referral Centre for Rare Lung Diseases, A. O. U. “Policlinico-Vittorio Emanuele” Dept. of Clinical and Experimental Medicine, University of Catania, 95123 Catania, Italy; 3 Artroreuma S.R.L., Outpatient of Rheumatology associated with the National Health System, Corso S. Vito 53, 95030 Mascalucia (Catania), Italy

**Keywords:** lung diseases, interstitial, multidetector computed tomography, cryptogenic organizing pneumonia, organizing pneumonia

## Abstract

To evaluate the radiological findings in patients with cryptogenic organizing pneumonia (COP) before steroid treatment and their behavior after therapy, we retrospectively evaluated a total of 22 patients with a diagnosis of COP made by bronchoalveolar lavage (BAL), biopsy or clinical/radiological features, and the patients were followed between 2014 and 2018 at the hospital; the demographic data, symptoms, radiologic findings, diagnostic methods and treatment plans of patients were collected from patients’ hospital records. At least two CT scans of 22 patients (16 female and six men) were evaluated, the first one before starting steroid therapy and the others after therapy. At baseline CT scans, the most common radiological finding was the presence of consolidations (18/22 patients, 81.8%); ground-glass opacities were also very common (15/25, 68.1%). The other findings were as follows: nodules and masses (5/22, 22.7%), atoll sign (4/22, 18.1%), perilobular pattern (3/22, 13.6%) and parenchymal bands (3/22, 13.6%). Two patients had a significant relapse after reducing/interrupting therapy, while three had a complete resolution and are not currently under therapy (maintenance of clinical remission with no oral corticosteroid (OCS)). In High-resolution computed tomography (HRCT) scans after therapy, consolidations were still observable in seven patients (five in new areas of the lung-migratory infiltrates), while most of them disappeared, leaving a residual area of ground glass opacity in two patients. One patient had a residual of the perilobular pattern, with the disappearing of the other findings (consolidations and ground-glass opacities). Two patients developed a fibrosing pattern despite the therapy (9.5%). Cryptogenic organizing pneumonia tends to respond to oral corticosteroid treatment, but some patients may have a null or partial response. We highlight the behavior of this disease after proper therapy.

## 1. Introduction

Organizing Pneumonia (OP) is a clinical, radiological and histological pattern characterized by the presence of granulation tissue within the terminal or respiratory bronchioles, in the alveolar ducts and surrounding alveoli, associated with chronic inflammation of the remaining lung parenchyma [1]. OP may be secondary to many extra pulmonary conditions—such as infections [2], drug toxicity [3,4,5,6], radiotherapy [4,7,8], autoimmune diseases [9,10], oncologic conditions [11,12], or pulmonary conditions—pneumonia, vasculitis, and lung cancer; when not associated with certain pulmonary or extra pulmonary disorders, it may be labelled as “cryptogenic” [2].

According to different studies, OP accounts for 1.8% up to 13% of all Interstitial Lung Diseases (ILDs) [13,14,15]; the mean age at onset varies from 50 to 60 years and males and females are usually equally affected. There is a prevalence in non-smokers [16] and it is not clear if (and why) smoking could be considered as a protective factor [17].

The idiopathic form of OP has been called Cryptogenic Organizing Pneumonia (COP), and it has been classified as an acute/subacute Interstitial Lung Disease in the American Thoracic Society/European Respiratory Society (ATS/ERS) statement of 2013 [18]. The term COP was first used by Davidson in 1983 [14], even if previously the same pathological entity was known as “Bronchiolitis Obliterans Organizing Pneumonia” (BOOP), as named by Epler [1]. This name was thereafter put aside, due to the semantic similarity with the morphological pattern of bronchiolitis obliterans, which is a completely different entity [19,20].

COP presentation is similar to many pneumopathies; therefore, an accurate clinical evaluation—in order to exclude these conditions before a COP diagnosis—is mandatory for clinicians and radiologists [13]. 

Classic symptoms include flu-like illness (fever, cough, weakness) and dyspnea, which can be mild or severe (the latter is associated to a rapidly progressive disease). Diagnosis is often delayed because of these unspecific manifestations [18]. On physical examination, crackles are the most common abnormality found. There is no finger clubbing [16].

At pulmonary function tests (PFTs), the most common abnormality is a restrictive ventilatory defect, and diffusing capacity for carbon monoxide (DLCO) often is reduced [18], with a mild hypoxemia in more than 80% of patients [16]. An obstructive ventilatory defect is shown in a minority of patients (20%), usually smokers. 

Bronchoalveolar lavage (BAL) is helpful for the differential diagnosis and could be used to exclude other disorders. The imaging findings and BAL could be useful for patients with appropriate clinical presentation and for those whose transbronchial biopsy is negative or for whom a confirmatory biopsy cannot be performed [21]. A “mixed pattern”, with increased lymphocytes (20–40%), neutrophils (about 10%), and eosinophils (about 5%), is the most frequent combination found in COP disease [21].

At imaging, COP may show different patterns at the onset. For this reason, its diagnosis could be a challenge. Knowing the onset presentation of a disease, and how it can change in time and during therapy, is essential for radiologists to be able to recognize it and avoid misdiagnosis. A definitive diagnosis of organizing pneumonia cannot be made on the sole basis of clinical findings, but imaging alone may be sufficient for a diagnostic hypothesis of OP; the combination of a compatible clinical history and adequate HRCT signs may enable diagnosis in 80% of cases [20,22]. As reported by Baha A. et al., patients who were not diagnosed histo-pathologically were diagnosed according to clinical and radiological features with a multidisciplinary approach [23].

Therefore, the aim of this work is to evaluate the temporal evolution of the radiological signs of COP, to determine the prevalent baseline pattern and changes after therapy.

### 1.1. Radiologic Features 

**Figure 1:**
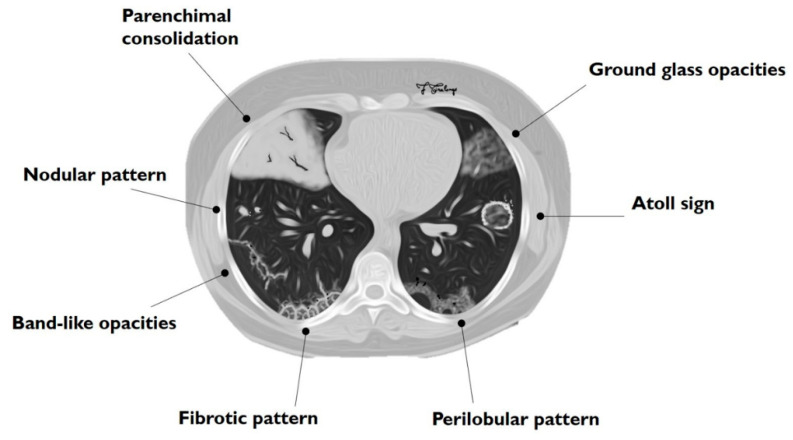
Schematic drawing of main imaging patterns of cryptogenic organizing pneumonia (COP).

The main HRCT findings in OP include: consolidation, ground-glass opacity (GGO), perilobular opacity, reversed halo opacity, nodules or masses, parenchymal bands, bronchial wall thickening, bronchial dilatation, mediastinal lymphadenopathy and pleural effusion [24,25]. The radiological features of COP can be divided into two main groups: typical and atypical patterns (Table 1, Figure 1). Typical pattern consists of multiple air spaces-opacities or peripheral consolidations with air bronchogram; as reported by Polverosi et al. [22], these consolidations may be limited by GGOs. According to many papers reported in literature, an atypical pattern includes: solitary or multiple nodules or masses (that constitute the nodular pattern), infiltrative opacities (infiltrative COP), atoll sign, bronchocentric lesions, linear and band-like opacities, a crazy paving pattern and progressive fibrosis [20,22,24,26].

### 1.2. Typical Pattern

**Figure 2:**
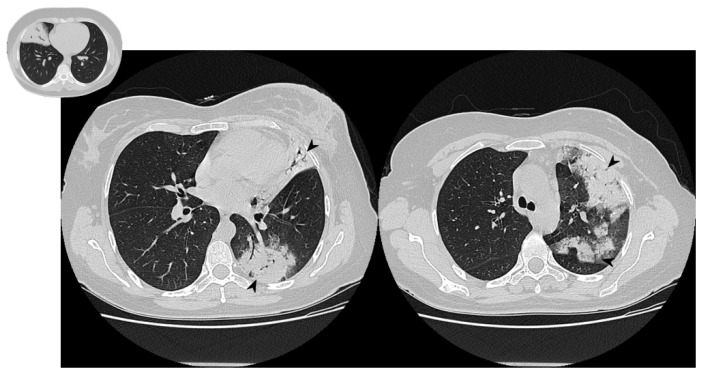
Typical pattern: multifocal and asymmetrical parenchymal consolidations (arrowheads), with peripheral distribution. These lesions may reproduce an air bronchogram sign in the context.

The classical form of COP is characterized by multifocal parenchymal consolidations, often bilateral and asymmetrical. These findings are usually described as patchy or with peripherical or peribronchial predominance in lower lobes. They tend to migrate, disappearing spontaneously and appearing in different sites (Figure 2) [22,26]. Consolidations may be associated with GGOs; usually, these lesions may reproduce an air bronchogram sign in the context [22]. At the moment of the onset, this typical pattern has been encountered in about 75% of patients [22,24,25,26].

The pattern represented by the presence of multifocal parenchymal consolidations should be considered a morphological presentation of other pathological conditions. In more detail, this appearance could be related to adenocarcinoma in situ, minimally invasive adenocarcinoma and invasive adenocarcinoma of the lung, primary pulmonary lymphoma, eosinophilic pneumonia, multifocal pneumonia, alveolar hemorrhage, multiple pulmonary infarctions, alveolar sarcoidosis, or ANCA-associated vasculitis [24]. If consolidations have a fluctuating presentation, the differential diagnosis can be reduced to four etiologies: OP, eosinophilic pneumonia, alveolar hemorrhages and vasculitis [26].

### 1.3. Atypical Pattern 

#### 1.3.1. Nodular Pattern

**Figure 3:**
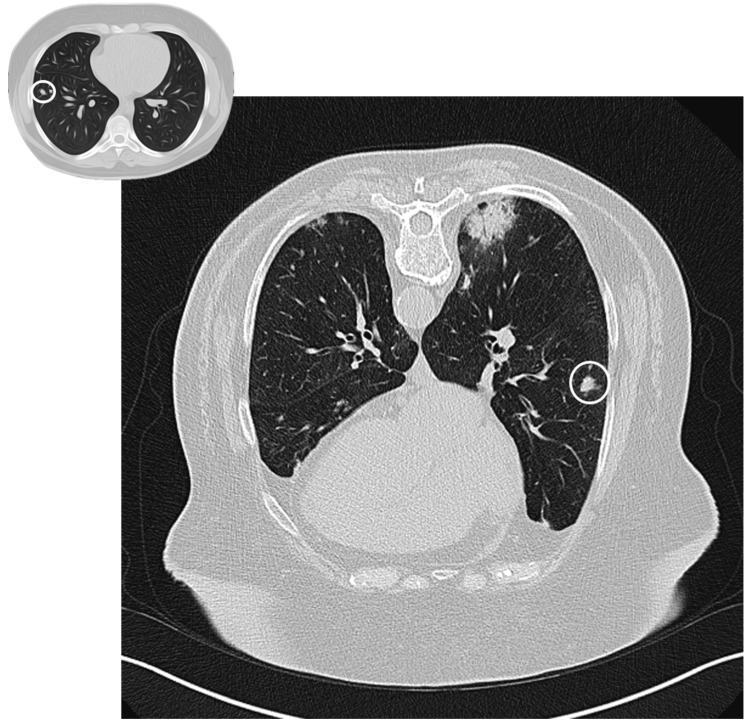
Nodular pattern: peripheral solid nodule (white circles) in a patient affected by COP.

A nodular pattern is seen in 15–50% of patients [25]. Nodules can be solid, part-solid or ground glass, with no specific distribution (sprinkled or peribronchovascular) (Figure 3); they could have spiculated borders, and, because of this appearance, the first suspect is often related to a malignant nature of lesions. If excavated (rare but possible), the nodule can mimic tuberculosis or septic embolism [24]. There are two nodular patterns: the well-defined “acinar” pattern (8 mm in diameter), or the ill-defined micronodular pattern (<4 mm) [20,26]. Acinar-type nodules represent focal areas of organizing pneumonia around plugged bronchioles and may have peribronchovascular or peripheral distribution. The micronodular pattern is an uncommon pattern of OP [20]; micronodulesmay have a peribronchial or centrilobular distribution, resembling a tree-in-bud pattern [26]. 

#### 1.3.2. Atoll Sign

**Figure 4:**
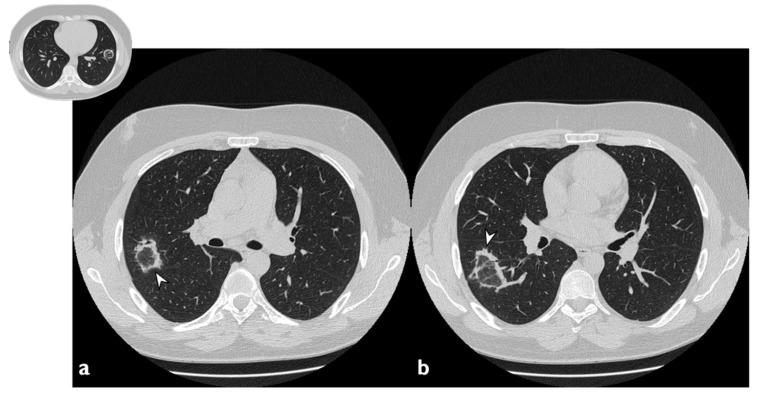
Atoll or reverse halo sign: areas of ground glass opacity surrounded by a ring- or a crescent-shaped consolidation (white arrowheads), which are clearly depicted in the right upper lobe (**a**) and in the right lower lobe (**b**).

The atoll sign (or reverse halo sign) was for a long time known to be pathognomonic of OP; in more detail, the term atoll was reported in literature in 1999 by Zompatori et al. [27], who described—in the cited paper—a ring-shaped opacity as a presentation of BOOP at HRCT. Actually, other conditions have been associated with this radiological feature: vasculitis, sarcoidosis with an atypical pattern, paracoccidiodomycosis, pneumocystis, tuberculosis, lipoid pneumonia and the complication of radio-frequency treatments. The atoll sign, however, has been classified among atypical patterns of COP, and it is related to the presence of an area of ground glass opacity surrounded by a ring-/crescent-shaped consolidation (Figure 4) [24,28,29]. 

#### 1.3.3. Crazy Paving Pattern

The crazy paving pattern—which is an uncommon presentation of COP—is represented by areas of ground-glass opacities superimposed to focal septal thickening of pulmonary parenchyma. It is a sign of different lung pathologies, such as infections, idiopathic interstitial pneumonias, Acute Respiratory Distress Syndrome (ARDS) and sarcoidosis [30,31,32].

#### 1.3.4. Progressive Fibrosis Pattern

**Figure 5:**
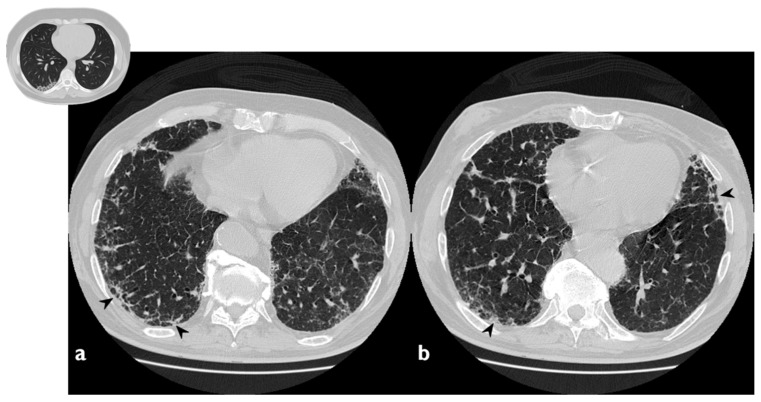
Fibrotic pattern: bilateral sub-pleural reticulations (arrowheads) and architectural distortion, clearly visible in peripheral regions of right lung (**a**) and left lung (**b**).

OP can produce sub-pleural basal reticulations and architectural distortion in about a 15% of cases, mimicking the appearance of nonspecific interstitial pneumonia (NSIP); the reticulations coexist with regions of consolidation or appear later (Figure 5) [24]. This presentation seems to be associated with a poor outcome [26]. As reported by OIkonomou et al., this pattern may occasionally result in honeycombing and must be differentiated from usual interstitial pneumonia (UIP) [20]. Pathologically, alveolar epithelial damage is a common condition in both OP and UIP. In OP, the necrosis of alveolar epithelium is followed by the migration of fibroblasts from the interstitial compartment to airways, while, in UIP, fibroblastic foci are restricted to interstitium, and intraluminal fibrosis is less extensive than in OP [20].

### 1.4. Perilobular Pattern

**Figure 6:**
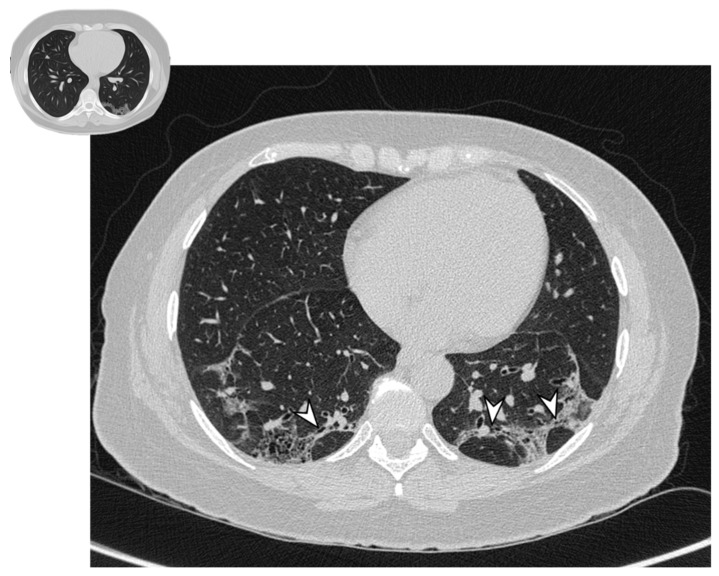
Perilobular pattern: arcade-like bands of parenchymal consolidation (white arrowheads) with blurred borders and thickening of the interlobular septa with a reticular pattern.

The perilobular pattern is characterized by curved or arcade-like bands of parenchymal consolidation with blurred borders and thickening of the interlobular septa—resembling a Roman Arch (Figure 6) [22]. It is a perilobular pattern and often associated with other opacities, especially consolidations [17,24]. The presence of air bronchogram sign helps to differentiate this pattern from atelectasis and fibrotic bands; other conditions that must be excluded are post-primary tuberculosis, lymphomatoid granulomatosis, lymphangitic carcinomatosis, fungal infections, sarcoidosis, pulmonary infarction and interstitial edema [33].

### 1.5. Linear and Band-Like Opacities

**Figure 7:**
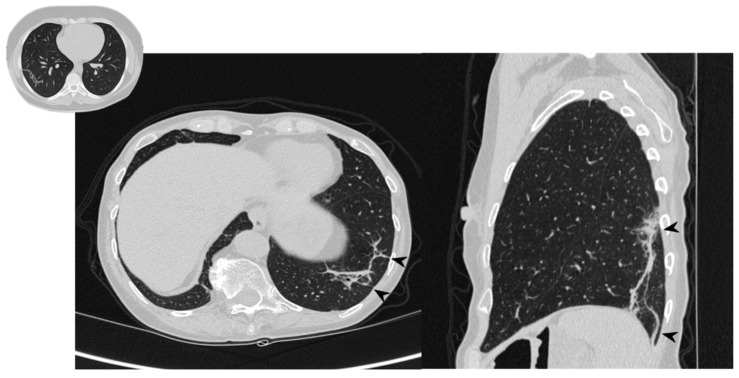
Linear and band-like opacities: sub-pleural curvilinear bands, parallel to the pleural (arrowheads) in a patient with COP.

This pattern is characterized by the presence of thick radial bands of consolidation containing an air bronchogram (feature that should be considered for a differential diagnosis with atelectasis) or sub-pleural curvilinear bands, parallel to the pleural (Figure 7). These imaging findings are very evocative of OP [24]. Linear abnormalities are often associated with other conditions, such as pulmonary edema, lymphangitic carcinomatosis, atelectasis and asbestosis [33].

### 1.6. Extra Pulmonary Radiological Findings

#### 1.6.1. Mediastinal Lymphadenopathy

Mediastinal nodes with a short-axis diameter > 1 cm are observed in 20–30% of patients with COP. The most frequently involved stations are lower paratracheal (station 4), subcarinal (station 7) and pulmonary hilum (station 10) (the stations’ number according to the International Association for the Study of Lung Cancer (IASLC) lymph node map [34]).

#### 1.6.2. Pleural Effusion

Small amounts of pleural effusion are seen in 10–35% of patients, usually bilateral [25].

## 2. Materials and Methods

### 2.1. Study Design, Setting, and Participants

We have selected patients who were assessed at our Regional Centre for Interstitial and Rare Lung Disease and Radiology Unit I of our Hospital (University Hospital “Policlinico-Vittorio Emanuele”, University of Catania) from January 2014 to April 2018 with a diagnosis of COP. 

The inclusion criteria were the following:

- A COP diagnosis, between January 2014 and April 2018, defined in agreement to the Update of the International Multidisciplinary Classification of the Idiopathic Interstitial Pneumonias [17], and performed in a multidisciplinary meeting on the basis of clinical data, laboratory tests, imaging features and, if a biopsy was performed, on the histological result;
- At least two HRCT scans, one at the baseline and one as 11–13 months follow-up;
- Availability of functional pulmonary tests at the baseline.

We have consulted the medical records and collected drug history, medical history and functional pulmonary tests. 

We have then excluded patients who have received a diagnosis of cancer within the previous 5 years, who underwent chest radiotherapy in the previous 5 years and patients affected by active or chronic infectious disease, active inflammatory diseases, autoimmune disorders or immunodepression. 

Our population study finally included 22 patients; for 4 patients having a clinical history of cancer, it should be noted that they received last treatment more than 5 years earlier. In particular, 1 received chemotherapy for follicular lymphoma, 1 received chemotherapy for colorectal cancer and 2 received radiotherapy for colorectal cancer. 

All patients have provided their informed consent before HRCT imaging at the time of the examination, and images were anonymized for research purpose

### 2.2. Protocol Details

The following eligibility criteria were adopted: patients with COP diagnosed according to the American Thoracic Society/European Respiratory Society/criteria [18], the availability of a complete clinical history, at least one-year clinical follow-up, at least two HRCT scans, at least two complete PFTs; in our institute, all diagnoses of interstitial lung diseases are routinely discussed in the universe of multidisciplinary meetings. We excluded patients with a history of chronic infections (viral, bacterial, or fungal) and those with advanced pulmonary fibrosis. We selected two CT scans: the baseline CT acquired at the diagnosis, and follow-up CT performed between 11 months and 13 months after the onset of disease. Radiological exams were evaluated in consensus by two radiologists with proven experience in pulmonary interstitial pathology, blinded to clinical or functional data. Parenchymal alterations were established according to the Fleischner Society Glossary of Terms for Thoracic Imaging [35] and were distinguished according to typicality and atypicality criteria. The extension and distribution of consolidations, ground-glass areas, atoll sign, crazy paving, fibrosis, perilobular abnormalities and parenchymal bands, the presence of pleural effusion, pleural thickening, pericardial effusion, and lymphadenopathy were recorded. Pattern changes and disease evolution after a one-year follow-up have been assessed, considering three possible scenarios: complete resolution, partial resolution and no resolution. The presence of new manifestations was also reported as “new ones”.

### 2.3. PTF—Pulmonary Function Test

All PFTs were performed according the ATS/ERS guidelines [36] at the Centre for Interstitial and Rare Lung Disease of our University Hospital, and included the following values: forced vital capacity (FVC), FVC%, forced expiratory volume (FEV), FEV1%, FEV1/FVC, diffusing capacity for carbon monoxide (DLCO) %. For each patient, the “Jaeger Vyntus Pneumo” manufactured by CareFusion (Carefusion, San Diego, CA, USA) was used. FVC and DLCO were expressed in Liters and as a percentage of the predicted value based on height, age, sex, and ethnic origin.

### 2.4. Image Acquisition

Since not all patients performed the first imaging tests in our Radiology Institute, we only considered chest CT scans that were compatible with HRCT standard protocol [37]. All the second imaging examinations were made at our Institute using a GE Optima CT660, manufactured by General Electric (GE Healthcare, Milwaukee, WI, USA). The following HRCT scanner parameters were used: kVp = 120–140; mA = automatic modulation dose; thickness = from 1.0 mm up to 1.25 mm; interval = overlapped images. Images were obtained throughout the thorax, covering from the bottom of the lungs to the lung apices, with sharp kernel imaging reconstruction. No contrast medium was administered in our series. 

### 2.5. Statistical Analysis

The data were collected on the Microsoft Excel database (Microsoft Corporate, Redmond, WA, USA) and analyzed using MedCalc program (MedCalc Software Ltd., Ostend, Belgium). We performed only a descriptive analysis for this study, reporting mean values, standard deviations and the percentage of distributions of clinical and morphological data; no statistical comparisons were performed, due to a limited number of cases. Continuous variables were presented as means (standard deviations), and categorical variables were presented as percentages. The authors had full access to the data and take full responsibility for its integrity. For this retrospective analysis, it was not necessary to request authorization from our ethics committee. The contents of this paper are consistent with the principles of the Declaration of Helsinki in the latest version.

## 3. Results

Based on the eligibility and exclusion criteria, we retrospectively enrolled a total of twenty-two patients (male: 6, age 58 ± 14 y.o.) with a diagnosis of COP. A histologic diagnosis was obtained in seven patients, an exfoliative cytology was obtained in one patient, and a positive bronchoalveolar lavage (BAL) was evaluated in six patients. For all other patients, the COP diagnosis was performed in the multidisciplinary field on the basis of clinical and radiological data.

In our sample, two patients were currently smokers and three were ex-smokers; four were oncologic patients (two underwent chemotherapy and two underwent radiotherapy). The most common symptoms were dyspnea and cough (dry cough was more frequent than productive), observed in 54.5% of patients. On physical examination, crackles were the most common finding. The demographic data and clinical features are summarized in Table 2. At PFTs, all the patients had a restrictive pattern, with a forced vital capacity (FVC) % value of 95 ± 24.8, and DLCO was reduced in 57% of patients. The results are summarized in Table 3. 

### 3.1. Imaging

#### Baseline CTs

In the initial CT scans, the most common radiological finding was the presence of consolidations, observed in 18 out of 22 patients (81.8%); in eight of these cases, patients had multiple consolidations (in the same lung or bilateral). Ground-glass opacities were also very common (15/22, 68.1%). Other findings were: nodules and masses (5/22, 22.7%), atoll sign (4/22, 18.1%), perilobular pattern (3/22, 13.6%) and parenchymal bands (3/22, 13.6%) Four patients had also mediastinal lymphadenopathy. These results have been summarized in Table 4.

### 3.2. HRCT Scans after Therapy

#### Consolidation

**Figure 8:**
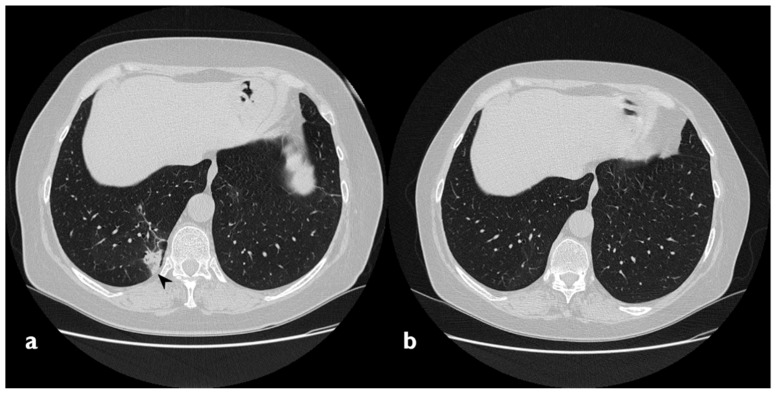
On the left: baseline HRCT (**a**); subpleural consolidation in the right lower lobe. On the right (**b**): HRCT after 11 months of steroid therapy shows a disappearance of the consolidation.

**Figure 9:**
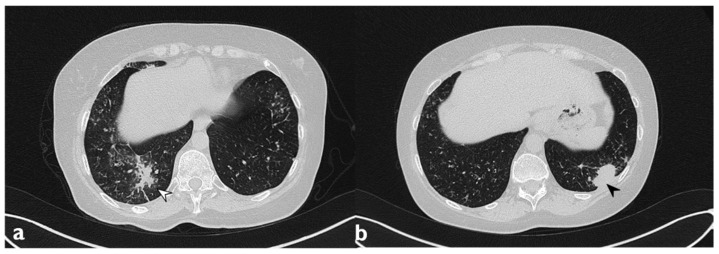
Migratory infiltrates. On the left (**a**): baseline HRCT; subpleural consolidation in the right lower lobe (white arrowhead). On the right (**b**): HRCT after 13 months of steroid therapy; the consolidations have disappeared in the first site, appearing in the left lower lobe (arrowhead).

In 14 of the 18 patients who presented consolidations at the first CT scan, a complete resolution of these consolidations was observed (Figure 8); however, in three of them, new ones showed up in different pulmonary areas (migratory infiltrates) (Figure 9). In the other four patients with consolidations on initial CT images, two patients had a partial resolution, whereas the remaining two cases have shown no differences in consolidations at follow-up HRCT. The changing consolidations are summarized in Table 5.

### 3.3. Ground Glass

**Figure 10:**
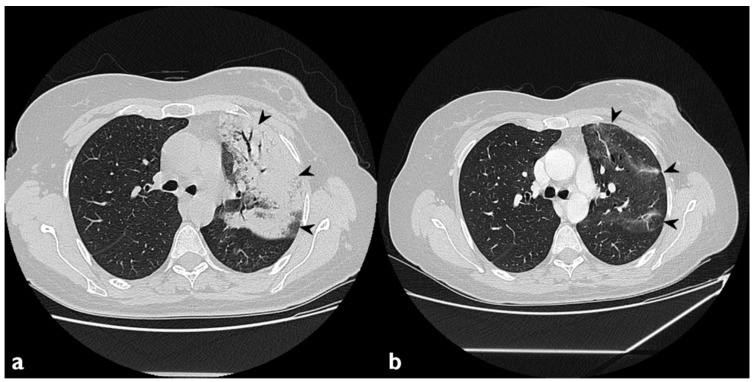
On the left (**a**): baseline HRCT; left parenchymal consolidations with air bronchogram sign in the context (arrowheads). On the right (**b**): HRCT after steroid treatment; new ground-glass areas (arrowheads) have appeared in the site of consolidations.

Ground-glass opacities completely disappeared in nine out of 15 patients that have shown this pattern at baseline HRCT. In two patients, new ground-glass areas appeared where a previous consolidation was present, like residual components of the consolidations (Figure 10) (Table 6).

### 3.4. Nodular Pattern

**Figure 11:**
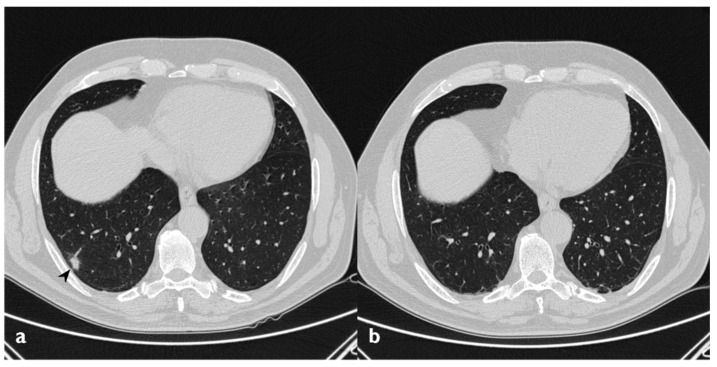
On the left (**a**): baseline HRCT; peripheral solid nodule in the right lower lobe (arrowheads). On the right (**b**): HRCT after oral corticosteroid (OCS) therapy; the previous nodule is no longer present.

**Figure 12:**
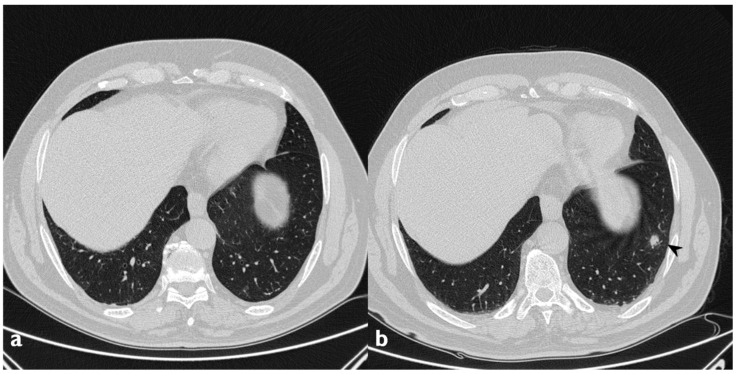
On the left (**a**): baseline HRCT. On the right (**b**): follow-up HRCT; a new nodule (arrowhead) appeared in the left lower lobe.

A complete resolution after therapy was observed in three of the five cases with nodular pattern (Figure 11), whereas two subjects have shown a partial reduction in number and size. Only one patient did not report nodule resolution after treatment. In one patient—who did not show nodules or masses at the first CT—we have observed the presence of new nodular lesions despite therapy (Figure 12) (Table 7).

### 3.5. Atoll Sign

**Figure 13:**
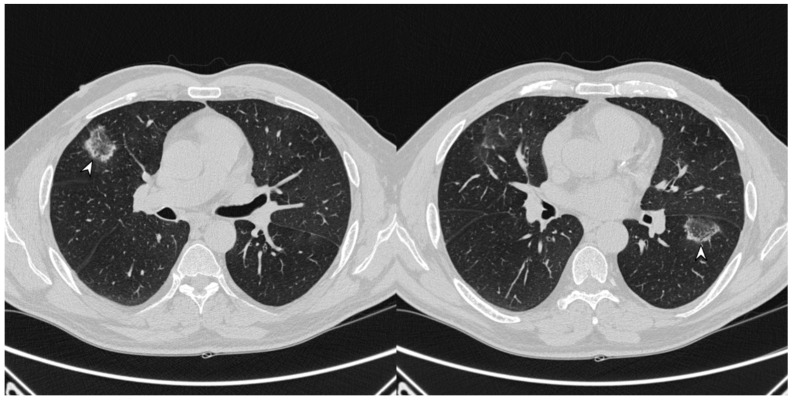
Presence of atoll signs (white arrowheads) in a patient with COP, which were still recognizable despite steroid therapy.

Of all four atoll signs, only one has been observed at follow-up HRCTs (Figure 13, Table 8).

### 3.6. PeriLobular Pattern 

**Figure 14:**
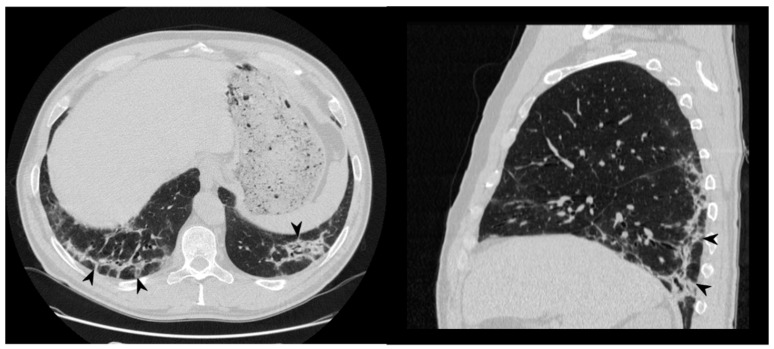
On the left: axial view. On the right: sagittal view. Perilobular pattern: the presence of arcade-like bands with blurred borders and thickening of the interlobular septa—resembling a Roman Arch (arrowheads), despite steroid therapy.

One patient—who has shown a perilobular pattern associated with other imaging findings (consolidations and ground-glass opacities)—maintained at follow-up only a residual appearance of this imaging finding. In more detail, a perilobular opacity was still depicted at follow-up CT—with the disappearing of the others (Figure 14). In the other two patients having a perilobular pattern (with a “Roman arch” sign), this imaging appearance was still visible at follow-up HRCT (Table 9), with no modifications.

### 3.7. Linear and Band-Like Opacities

**Figure 15:**
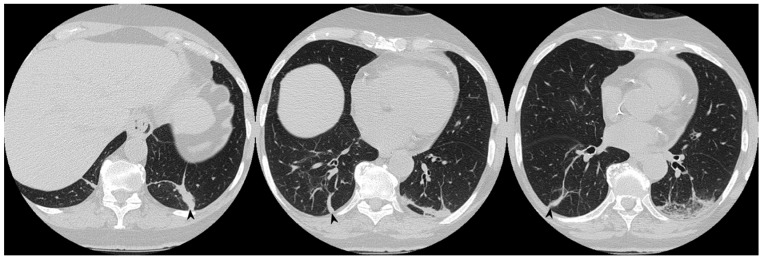
Persistence of band-like opacities (arrowheads) despite 13 months of steroid treatment.

Linear and band-like opacities were still observable in one patient out of three (Figure 15), but a new linear opacity appeared in one patient who did not show it in the first CT (Table 10).

### 3.8. Evolution and Clinical Outcome 

**Figure 16:**
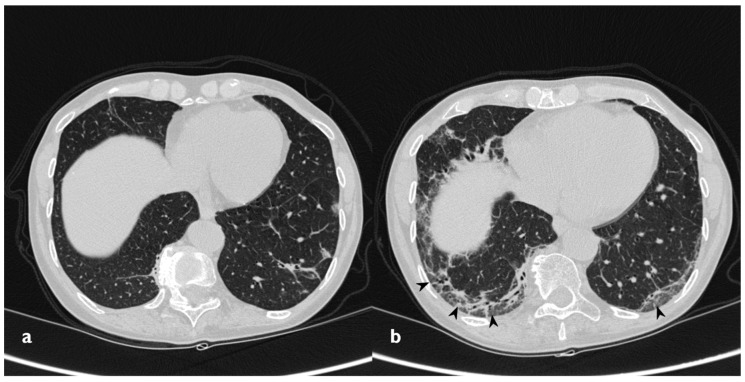
Fibrosing evolution. On the left (**a**): baseline HRCT. On the right (**b**): HRCT after 11 months of steroid therapy; new signs of fibrosis-sub-pleural basal reticulations and architectural distortion (arrowheads).

Two patients developed a fibrosing pattern despite the therapy (Figure 16). In more detail, in these two cases, we have observed multiple consolidations and ground-glass opacities at the beginning, with the partial resolution of these findings at follow-up HRCTs (the disappearance of ground-glass opacities and partial reduction of consolidations); however, they developed fibrotic changes like basal reticulations, bronchiectasis and fibrotic striae. Twenty-one patients (95.4%) received steroid therapy (oral corticosteroid, OCS) during the observation period of the study, and 17 of them had regression of radiological findings. Two patients had significant relapse after reducing/interrupting therapy (Table 11); both of them showed a typical consolidative picture at baseline. In one patient, relapse was characterized by the increase in multiple consolidations, particularly in the left lower lobe. This patient presented dry cough, dyspnea, crackles and inspiratory squawks, particularly in the left lower lobe. SpO2 level was 98%. In the other patient, the relapse was characterized by the appearance of multiple consolidations with air bronchogram. This patient presented exertional dyspnea, productive cough and important asthenia. SpO2 level was 98%. One patient refused steroid therapy and was followed up without any immunosuppressive therapy; for this patient, we did not observe any significant deterioration of functional data and radiological features. 

## 4. Discussion

COP is a cryptogenic disease with motley modalities of presentation. In our study, the mean age of onset was 58 years, and patients were aged between 25 and 84 years; a predominance in women (68%) was observed. These data are in line with the study of Niksarlioglu et al. [38] and the study of Lee et al. [39]. COP is usually a no smoke-related disease; our results confirm this data, as seen also in Niksarlioglu’s and Lee’s studies, with only five patients currently or ex-smokers (80.8% non-smokers). For what concern symptoms, we observed dyspnea and cough as the most common symptoms (54.5% both symptoms). On physical examination, crackles were found in four patients (18.2%), inspiratory squawks in one patient and expiratory wheeze in two patients. In comparison to Sveinsson’s study of 2007 [40], where fever was present in 50% of patients, in our study, only 13.6% of patients complained this symptom. In Niksarlioglu et al.’s study, cough was the most common symptom (88.2% of patients), while 54.5% of our patients experienced cough (in the majority of case it was dry cough) [38].

### 4.1. PTFs

The most common abnormalities consisted of restrictive ventilator defect at spirometry and reduced DLCO [13]; these reports agree with our data. Niksarlioglu et al. have described that “an obstructive pattern may be observed in smokers (…) and an obstructive pattern was determined in one patient who was a smoker” [38]; however, in our study, we did not observe any obstructive pattern.

### 4.2. Radiological Finding

In our population, a typical imaging pattern of disease was the most common onset pattern on HRCT; in more than 80% of patients, consolidations were bilateral, asymmetrical and migrating. These results are in line with previous papers published by Faria et al. (who reported typical pattern in 83% patients) [41] and by Lee et al. (who reported typical pattern in 77% patients) [39]. The second most prevalent radiological pattern was represented by GGOs; in our study, we observed GGOs in 68.1% of patients, unlike what has been reported in previous studies about this subject, where patients have shown GGOs in a range between 86% and 89% [36,38]. The atoll sign was found in four patients (18.1%), similar to the description of Kim et al. in their study of 2003 [29].

### 4.3. Outcome

A total of 21 of 22 patients received OCS therapy, with following improvement in clinical findings. Two patients (9.1%) had significant relapse after reducing/interrupting therapy, and this behavior respects what was already reported in previous articles [38,39]. 

Two patients (9.1%) are currently no more under therapy (the maintenance of clinical remission with no OCS). In total, 86.9% of patients are under maintenance therapy with a low dose of OCS. Only one patient refused therapy.

## 5. Limits

As similar papers already reported in the literature, this study is limited because of a retrospective design and a relatively small population; in addition, the main limitation of this paper is represented by the small number of patients having histopathological diagnosis (only seven out of 22 cases)—even if all cases have been discussed and evaluated using a multidisciplinary approach that, in some cases, could define when biopsy is needed, as reported in the Update of the International Multidisciplinary Classification of the Idiopathic Interstitial Pneumonias [18]. 

In addition, the lack of histological data is partially related to the observational retrospective design of our study; in more detail, for patients without a biopsy-proven diagnosis, we have considered typical clinical features and radiological findings observed in the clinical history or data of exfoliative cytology and bronchoalveolar lavage, combined with clinical and radiological features, as reported in previous papers by Baha et al., Oiknomou et al. and Jara-Palomares et al. [20,21,22,23]. Moreover, the lack of histological samples could be partially explained by the fact that some patients did not accept biopsy once they had gradually experienced an improvement in their symptoms. 

However, disease progression in patients with atypical patterns and responsiveness to therapeutic treatment has been described only in a few research articles and in small populations; these factors still represent fascinating topics, meaning that it could be useful to analyze a COP population through a prospective multidisciplinary all approach—providing an accurate collection of clinical, radiological and morphological data. 

Finally, in our study, adherence to therapy was ascertained only on the basis of what was stated by patients, but this limit is common to all studies involving home therapy.

## 6. Conclusions

In our series, COP was more prevalent in middle-aged female and no-smoker; dry cough and dyspnea were the most common symptoms in these patients. According to what was already reported in previous literature articles, COP having typical imaging features was the most common onset pattern, with consolidations and ground-glass opacities frequently recognized on HRCT images. However, partial resolution and some changes may be observed during the follow-up, with the possibility of fibrotic abnormalities found in a small percentage of cases.

## Figures and Tables

**Table 1:** Patterns of COP.

Typical Pattern	Atypical Patterns
Multiple alveolar opacities	Nodular pattern
	Perilobular pattern
	Crazy paving pattern
	Progressive fibrosis COP
	Atoll sign
	Linear and band-like opacities

**Table 2:** Demographic data of our population study.

Variables	Patients Number (%)
N° of patients	22
Age (in years)	58, range 25–84
Sex	15 F (68.1%)/6 M (27.2%)
Smoking	5 (22.7%)
Ex-smokers	3 (60%)
Currently smokers	2 (40%)
Chemotherapy	2 (9%)
Radiotherapy	3 (13.6%)
Rheumatoid Arthritis	1 (4.5%)

**Table 3:** Clinical and functional features. FVC: forced vital capacity, FEV1: Forced expiratory volume in 1 second, DLCO: diffusion lung carbon monoxide.

Symptoms	Patients Number (%)
Dyspnea	12 (54.5%)
Cough	12 (54.5%)
Dry	10 (83.3%)
Productive	2 (16.7%)
Weight loss	1 (4.5%)
Weakness	4 (18.2%)
Pleural effusion	2 (9.1%)
Fever	3 (13.6%)
Chest pain	1 (4.5%)
Crackles	4 (18.2%)
Inspiratory squawks	1 (4.5%)
Expiratory wheeze	2 (9.1%)
**PFT**	**Values**
FVC (L)	2.9 ± 1.0
FVC (%)	95 ± 24.8
FEV1 (L)	3.2 ± 3.5
FEV1 (%)	93.8 ± 23.3
FEV/FVC	93.4 ± 12.8
DLCO (%)	84.5 ± 13.6

**Table 4:** Most common HRCT findings at baseline.

HRCT Findings	Number of Patients (%)
Consolidation	18 (81.8%)
GG opacities	15 (68.1%)
Atoll sign	4 (18.1%)
Nodular pattern	5 (22.7%)
Perilobular pattern	3 (13.6%)
Linear and band-like opacities	3 (13.6%)
Mediastinum lymphadenomegaly	4 (18.1%)

**Table 5:** Evolution of the 18 patients who presented consolidations.

Evolution of Consolidations	Number of Patients (%)
Complete resolution	11 (61.1)
Complete resolution + new ones	3 (16.6)
Partial resolution	2 (11.1)
No resolution	2 (11.1)

**Table 6:** Evolution of the 15 patients who presented ground-glassopacities.

Evolution of Ground-Glass Opacities	Number of Patients (%)
Complete resolution	9 (60)
No resolution	6 (40)
New ones	2

**Table 7:** Evolution of the 5 patients with a nodular pattern.

Evolution of Nodular Pattern	Number of Patients (%)
Complete resolution	3 (60)
Partial resolution	2 (40)
No resolution	1 (20)
New ones	1

**Table 8:** Evolution of the 4 patients with atoll sign.

Evolution of Atoll Sign	Number of Patients (%)
Complete resolution	3 (75)
No resolution	1 (25)

**Table 9:** Evolution of the 3 patients with perilobular pattern.

Evolution of Perilobular Pattern	Number of Patients (%)
Complete resolution	0
No resolution	3 (100)

**Table 10:** Evolution of the 3 patients with linear and band-like opacities.

Evolution of Linear and Band-Like Opacities	Number of Patients (%)
Complete resolution	2 (67)
No resolution	1 (33)
New ones	1

**Table 11:** Different kinds of evolution of the 21 patients who received steroid treatment.

Evolution	Number of Patients (%)
Regression of radiological findings:	17 (80)
Complete (no maintenance therapy)	2 (9.5)
Significant relapse	3 (14.2)
Fibrosing evolution	2 (9.5)
